# Percutaneous Pulmonary Valve Implantation In Native Right Ventricular Outflow Tract Using Myval™ Without Pre‐Stenting

**DOI:** 10.1002/hsr2.70598

**Published:** 2025-04-09

**Authors:** João Luiz Langer Manica, Raul Ivo Rossi Filho, Carlos Augusto Cardoso Pedra, Fabio Vieira Caovilla, Francisco Chamié, Carlo Benatti Pilla, Pablo Thomé, Vinicius Fraga, Marcelo Ribeiro, Germana Cerqueira Coimbra, João Henrique Aramayo Rossi, Ênio Guerios, João Vitor Slaviero, Santiago Raul Arrieta

**Affiliations:** ^1^ Instituto de Cardiologia Porto Alegre Brazil; ^2^ Hospital Moinhos de Vento de Porto Alegre Porto Alegre Brazil; ^3^ Instituto Dante Pazzanese de Cardiologia São Paulo Brazil; ^4^ INTERCAT – Cardiologia Intervencionista Rio de Janeiro Brazil; ^5^ Santa Casa de Misericórdia de Porto Alegre Porto Alegre Brazil; ^6^ Hospital Unimed Piracicaba Piracicaba Brazil; ^7^ Hospital Unimed Vitória Vitória Brazil; ^8^ Universidade de São Paulo—Instituto do Coração, Incor São Paulo Brazil; ^9^ Hospital Albert Einstein São Paulo Brazil; ^10^ Universidade Federal do Paraná Hospital de Clínicas Curitiba Brazil

**Keywords:** percutaneous, pulmonary regurgitation, pulmonary valve implantation, right ventricular outflow tract, tetralogy of fallot, transcatheter

## Abstract

**Background and Aims:**

Percutaneous pulmonary valve implantation was introduced two decades ago as a low‐risk alternative for patients previously operated with conduits presenting RVOT dysfunction. Recently, Sapien valve was approved for use in the pulmonary position but this valve cannot be used for RVOT larger than 29 mm. Self‐expanding valves can be used for large native RVOT, however, there are some cases not suitable for this technique. The advent of 30.5 and 32 mm Myval™ balloon expandable heart valves provided the possibility to treat these patients percutaneously. The objective of this study is to describe immediate results of 30.5 and 32 mm MyVal™ valves implantation in native and large RVOT without pre‐stenting.

**Results:**

Seventeen patients underwent to percutaneous pulmonary valve implantation to treat large and native dysfunctional RVOT. The mean patient age was 26.2 ± 13.0 years, and the mean patient weight was 64.8 ± 14.8 kg. All patients had been previously submitted to at least one surgical procedure. The implantation was directly performed without pre‐stenting in all patients. The mean basal RVOT diameter was 27.6 ± 2.1 mm. Five patients underwent to 30.5 mm and 12 patients underwent to 32 mm pulmonary valve implantation. All patients had satisfactory valve function immediately after valve implantation. The average hospital stay was 3.2 days.

**Conclusion:**

In conclusion, the 30.5 and 32 mm MyVal™ valves have significant potential for treating patients with large native RVOTs, providing a safe and effective alternative to traditional surgical approaches and existing percutaneous options.

**Abbreviations**
PPVI: percutaneous pulmonary valve implantation
RVOT: right ventricular outflow tract

## 1 Introduction

Percutaneous pulmonary valve implantation (PPVI) was first introduced in 2000 as a minimally invasive alternative for patients with right ventricular outflow tract (RVOT) dysfunction following surgical repair. Initially, PPVI procedures used the Melody™ Valve (Medtronic, Minneapolis, USA) [1], which was originally designed for use in surgical conduits. Its application was extended to other anatomical configurations with landing zones up to 24 mm. Development of the Edwards Sapien™ Valve (Edwards Life Sciences, Irvine, USA) allowed PPVI in RVOTs up to 29 mm in diameter [2]. Subsequently, self‐expandable valves, such as the Venus P™ (Venus Medtech, Shanghai, China), Harmony™ (Medtronic), and Alterra™ (Edwards Life Sciences), expanded the scope to native and large RVOTs exceeding 30 mm [3, 4]. However, specific anatomical configurations, such as pyramid‐shaped outflow tracts, are not suitable for self‐expandable valves due to their complex geometries [5].

In Brazil, the unavailability of self‐expandable valves significantly limits the options for percutaneous treatment in many patients with large native RVOTs. The introduction of the MyVal™ balloon‐expandable valve (Meril Life Sciences Pvt. Ltd., Vapi, Gujarat, India) with diameters of 30.5 and 32 mm provided a novel solution for patients with outflow tracts exceeding 30 mm and could be used “off‐label” in the pulmonary position [6]. The present study reports an initial experience implanting MyVal™ in large RVOTs and aimed to evaluate the feasibility and safety of this procedure without pre‐stenting, which could simplify the intervention and reduce associated risks.

## 2 Methodology

This is a multicenter registry study involving patients who underwent implantation of 30.5 mm and 32 mm MyVal™ valves in the RVOT without prior stent placement. Patients received intravenous prophylactic antibiotics and unfractionated heparin (100 units/kg) during the procedure, with additional doses targeting an activated clotting time of 250–350 s. To ensure comprehensive evaluation and successful implantation, each patient had three venous accesses and one arterial access.

**Figure 1:**
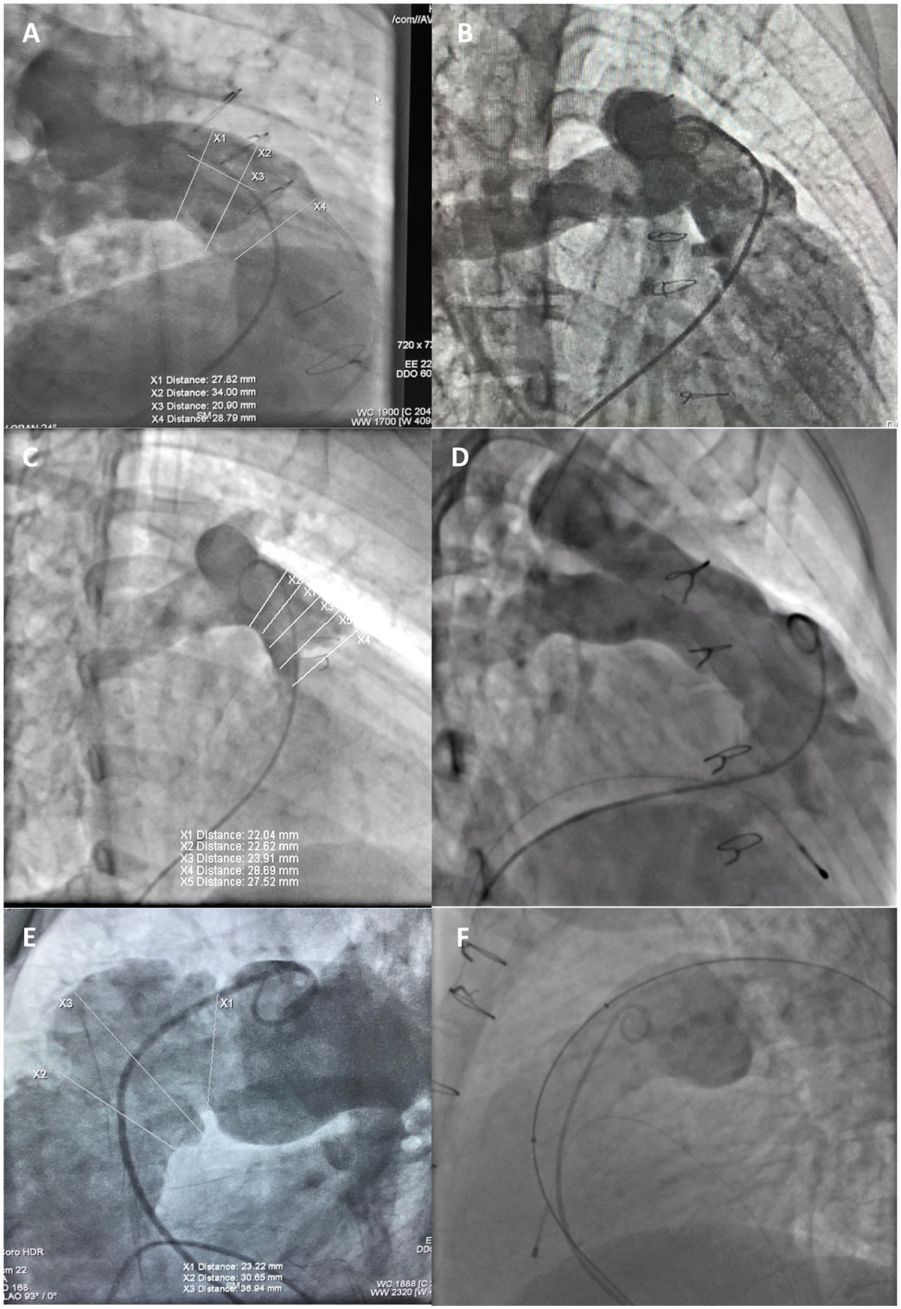
Preprocedural cranial angiographic assessments revealed distinct morphological variations: Cranial RAO demonstrated a tubular ectatic RVOT configuration (A); cranial RAO demonstrated a tubular RVOT with minimal pre‐bifurcation constriction (B); cranial RAO displayed a pyramidal‐shaped RVOT (C); cranial RAO exhibited a uniform tubular RVOT architecture (D); lateral 90° projection delineated a complex RVOT anatomy characterized by two distinct indentations (E) and another Lateral 90° projection outlined a tubular RVOT with markedly dilated Valsalva Sinuses (F). RVOT: right ventricular outflow tract; RAO: right anterior oblique.

**Figure 2:**
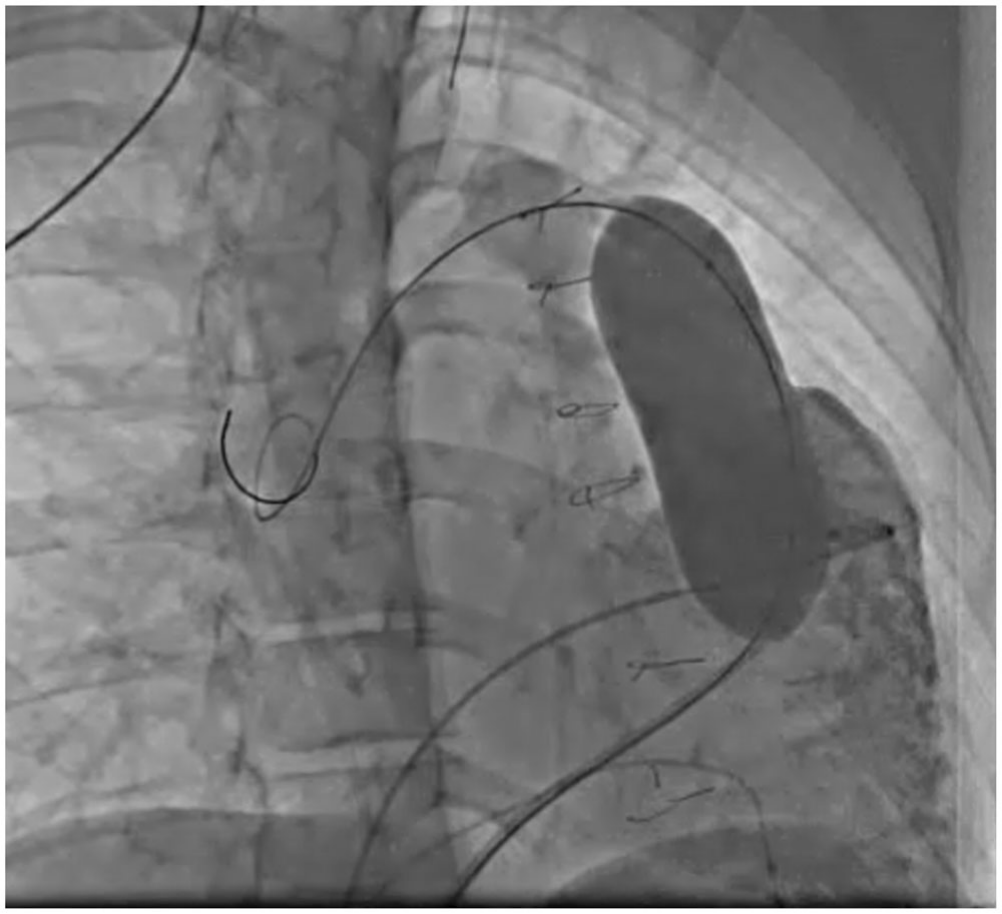
Complete 30 mm Cristal balloon inflation (Balt®, Montmorency, F in the right ventricular outflow tract with simultaneous right ventriculography under overdrive suppression of cardiac output by a pacemaker with a heart rate between 180 and 200 beats per minute.

Right ventriculography was performed in the right anterior oblique (RAO) (30°–40°) cranial (20°) and lateral (90°) views to assess the anatomy of the RVOT (Figure 1). To evaluate RVOT distensibility and coronary compression risk, complete 30 mm Cristal™ balloon (Balt, Montmorency, France) inflation was performed in the RVOT with simultaneous right ventriculography under overdrive pacing (180–200 bpm) to provide a clear image of the outflow tract (Figure 2).

At one referral center, all patients with ecstatic and borderline RVOTs up to 29 mm in diameter underwent extensive pre‐implantation computed tomography evaluation with three‐dimensional reconstruction and bench test with “in vitro” implantation of 32‐mm‐diameter valves to establish the indication for the procedure.

Patients with a stretched RVOT, with a diameter between 28 and 32 mm measured using a fully inflated 30 mm noncompliant balloon, were included in the study. Exclusion criteria were RVOT diameter < 28 mm, residual antegrade flow after complete balloon inflation, balloon instability, and apparent risk of coronary compression. The choice of valve diameter was based on the balloon indentation measurement: 30.5 mm valves were used for balloon indentation between 28 and 29 mm, whereas 32 mm valves were used for indentation > 29 mm. Patients in whom the stretched diameter of the balloon did not show clear indentation when fully inflated but remained stable without contrast leakage through the RVOT underwent implantation with a 32 mm valve. Additional volume (2–5 mL) was added to the balloon inflator to ensure proper apposition of the valve.

Myval™ is characterized by a nickel–cobalt alloy frame composed of a single element–hexagon arranged in a hybrid honeycomb fashion, sewn bovine pericardium leaflets. This unique structure of hybrid honeycomb allows 53% of the frame to have large open cells toward the pulmonary end and 47% of the frame to have closed cells with higher annular radial force toward the ventricular end. At this portion, an outline polyethylene terephthalate skirt exists in to avoid paravalvular leakage. Valve diameters range from 20 to 32 mm sizes in 1.5‐mm increments and the length of the valve is changing from 17 to 21 mm.

**Figure 3:**
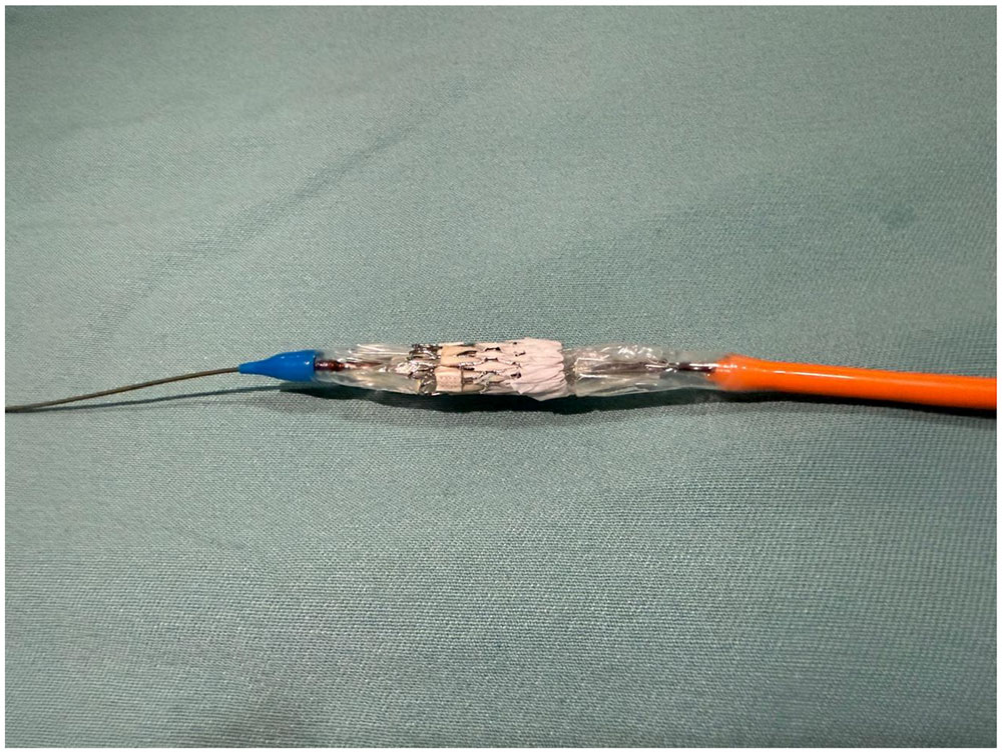
Transcatheter pulmonary valve replacement via a venous antegrade approach requires meticulous attention to leaflet orientation, achieved by inverting the valve ends during assembly onto the deflated balloon of the Navigator system comparing to transcatheter aortic valve replacement. The ventricular end, which includes the skirt and closed cells, must be aligned with the proximal stopper, while the pulmonary end, consisting of open cells, should be positioned at the distal end of the balloon to ensure optimal leaflet function and hemodynamics.

The MyVal™ bioprosthesis, which was initially designed for aortic valve implantation, was adapted for pulmonary use by inverting its ends during assembly (Figure 3). The proximal and distal internal inflation ports create a “dog‐bone” shape, providing stability and preventing embolization during navigation and implantation. The expandable hydrophilic 30 cm length Python™ introducer sheath has an inner diameter of 14 F and expands temporarily to allow the passage of a preassembled valve replacement. In addition to its low profile, the design of the sheath allows complete retrieval of the device in the IVC before it is inflated for later reinsertion into the patient through the same delivery system or abortion of the procedure [6, 7].

**Figure 4:**
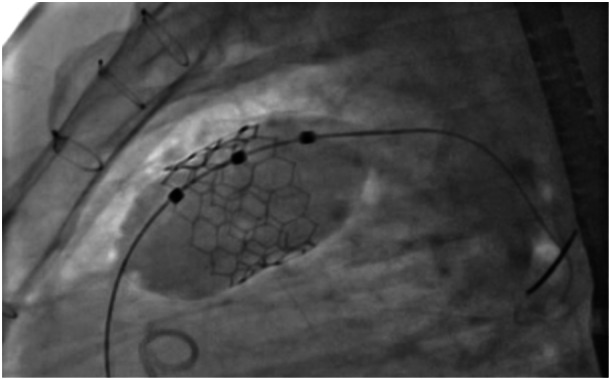
Direct 32 mm MyVal balloon expandable valve in an ectatic right ventricle outflow tract without previous stent implantation under overdrive suppression.

The primary objective of this study was to demonstrate the safety and efficacy of MyVal™ for implantation in large RVOTs without pre‐stenting (Figure 4). Adverse events, such as death, emergency surgery, device embolization, and coronary compression, were monitored. Efficacy was evaluated by echocardiography immediately after the procedure, focusing on the presence of trivial or mild pulmonary valve regurgitation and a transvalvular gradient < 20 mmHg.

The study adhered to international ethical standards, including the Helsinki Declaration. Informed consent was obtained from all patients or their guardians. The institutional review boards or ethics committees at participating centers approved publication of the data.

### 2.1 Statistical Analysis

Continuous variables are expressed as means with standard deviations. Categorical variables are presented as frequencies and percentages. Significance was determined using appropriate tests, such as the t‐test for continuous variables and chi‐squared for categorical variables, with *p* < 0.05 considered significant. Data were analyzed using statistical software to ensure accuracy and reliability (SPSS 30.0.0).

## 3 Results

Between June 2020 and January 2024, 17 patients (13 with transannular patch repair, 1 with bovine pericardial tube, 2 with homograft, and 1 with arterial switch) across 12 institutions in Brazil underwent pulmonary implantation of 30.5 and 32 mm MyVal™ valves without pre‐stenting. One author participated in 15 of the 17 procedures, all involving patients with at least one previous surgical intervention.

Demographic and procedural data are presented in Tables 1 and 2, respectively. The mean patient age was 26.2 ± 13.0 years, and the mean patient weight was 64.8 ± 14.8 kg. Fifteen patients (88%) had a diagnosis of tetralogy of Fallot. All were referred for PPVI due to pulmonary insufficiency.

**Table 1:** Demographic data.

Patient	Sex	Diagnosis	Age (years)	Weight (Kg)	No. of previous surgeries
1	F	ToF	56	75	1
2	F	ToF	18	66	2
3	M	ToF	32	74	1
4	M	ToF	46	90	1
5	F	ToF	21	85	3
6	M	ToF	16	54	2
7	F	ToF	27	68	1
8	M	ToF	41	71	1
9	F	ToF	15	40	2
10	F	ToF	44	45	1
11	M	ToF	18	60	2
12	M	ToF	24	60	2
13	M	Critical neonatal PVS	14	54	1
14	M	TGV	21	84	1
15	F	ToF	17	62	1
16	M	ToF	11	42	1
17	F	ToF	26	72	1

Abbreviations: PVS, pulmonary valve stenosis; T4F, tetralogy of Fallot; TGV, transposition of the great vessels.

**Table 2:** Procedural data.

Patient	RVOT diameter (mm)	Post‐balloon RVOT Diameter (mm)	MyVal diameter (mm)	Final diameter (mm)	Overexpansion rate (%)	No. of complications	Hospital stay (days)
1	28	30	32	32	0	0	2
2	28	29	30.5	30	0	0	2
3	25	29.5	32	32.5	1.5%	0	2
4	29	31.2	32	33.7	5.3%	0	3
5	29	30	32	32	0%	0	2
6	27.5	28	30.5	30.5	0%	0	2
7	32	32	32	34.7	8.4%	0	2
8	28	30	32	33.5	4.7%	0	2
9	27	28	30.5	30	0%	0	2
10	30	30	32	33.5	4.7%	1*	13
11	31	31	32	35	9.4%	0	2
12	27	30	32	30	0%	0	2
13	26	30	32	32	0%	0	2
14	24	28	30.5	30.5	0%	0	2
15	27	30	32	32	0%	0	2
16	26	28	30.5	30.5	0%	0	2
17	25	29	32	32	0%	1+	8

Abbreviation: RVOT: right ventricle outflow tract.

* Takotsubo syndrome due to inotropes. +Partial coronary compression without clinical significance.

The mean basal RVOT diameter was 27.6 ± 2.1 mm, and we found no significant difference among patients implanted with 30.5 mm versus 32 mm valves (27.5 mm vs. 27.9 mm, respectively, *p* > 0.05). After completing 30 mm Cristal balloon inflation, the mean diameter increased to 29.5 ± 1.2 mm. The valve implantation without pre‐stenting was successful in all patients. Five patients received 30.5 mm valves, all of them with stretched RVOT diameters between 28 and 29 mm (mean 28.2 ± 0.4 mm). Twelve patients with stretched RVOT diameters > 29 mm (mean 30.3 ± 0.9 mm) received 32 mm valves. The final valve diameter varied between 30 and 35 mm, customized according to RVOT size. The mean diameters for the 30.5 mm and 32 mm devices were 30.3 ± 0.2 and 32.7 ± 1.3 mm, respectively. No significant RVOT gradient (>, 15 mmHg) was observed postimplantation.

**Figure 5:**
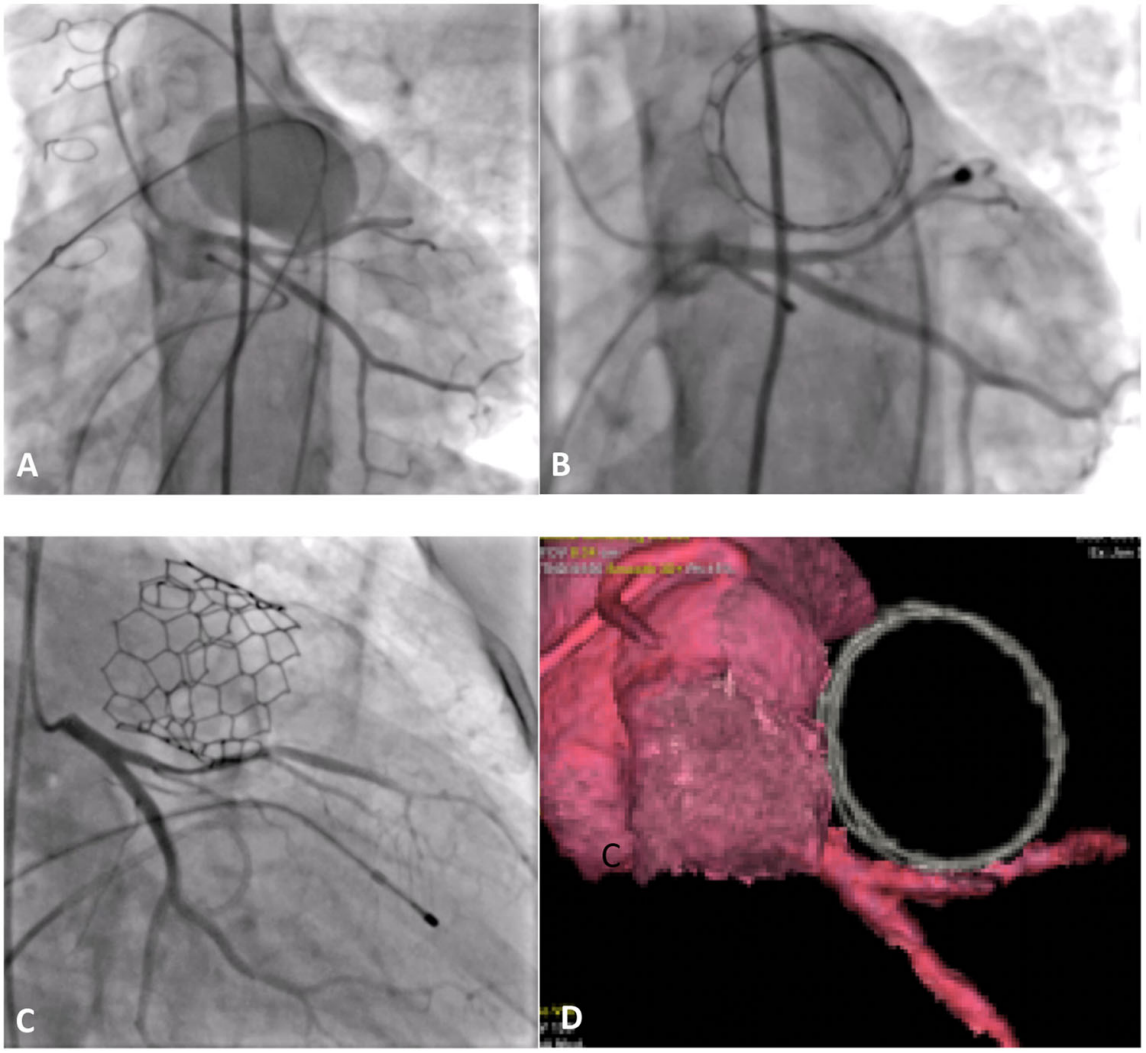
Crystal balloon interrogation in the RVOT with simultaneous left coronary artery angiography in a caudal left anterior oblique (LAO) projection (A). Left coronary artery angiography in the same projection after valve implantation, demonstrating partial coronary compression (B). Left coronary artery angiography in a cranial right anterior oblique (RAO) projection post‐valve implantation, also showing partial coronary compression (C). CT scan performed 48 h postprocedure demonstrating the valve's proximity to the coronary artery without evidence of obstruction (D). CT, computed tomography; LAO, left anterior oblique; RAO, right anterior oblique; RVOT, right ventricular outflow tract.

Hyperthermia occurred in five patients (23.5%) and resolved within 48 h without intervention. One patient experienced pulmonary bleeding pre‐implantation, requiring ventilation and vasoactive drugs; the valve was successfully implanted, but the patient presented signs of hemodynamic instability. Echocardiogram soon after being admitted to the ICU demonstrated Takotsubo syndrome, probably related to the use of metaraminol. Discontinuation of the vasoactive drug resulted in improvement of the patient, who was discharged 13 days later. One patient presented with partial (around 50%) anterior descending artery compression postimplantation after a 32 mm Myval implantation (Figure 5). This patient was submitted to myocardial functional stress tests during hospitalization, remaining asymptomatic with no signs of ischemia. No cases of arrhythmias, prosthesis embolization, or cardiovascular complications were reported.

Postprocedure echocardiograms showed a mean RVOT gradient of 11.4 ± 4.6 mmHg and minimal, if any, pulmonary insufficiency in 16 patients. Mild insufficiency was noted in one patient. The average hospital stay was 3.2 days (range 2–13 days), with all patients receiving antiplatelet monotherapy for at least 12 months postimplantation.

## 4 Discussion

Pulmonary regurgitation is a significant contributor to biventricular dysfunction, arrhythmias, and increased mortality. In patients with surgically repaired tetralogy of Fallot, chronic pulmonary regurgitation leads to progressive RVOT dilation, complicating PPVI [8, 9]. Reoperation poses surgical risks, whereas PPVI offers lower morbidity, faster recovery, and shorter hospital stays [10].

For RVOTs exceeding 30 mm, PPVI has been limited to self‐expandable valves, which are not available in Brazil [10]. Recent studies on self‐expandable valves, such as Venus P™, Harmony™, Pulsta™, and more recently Alterra™ Adaptive Prestent specifically designed to reduce the diameter of the RVOT, thereby creating an optimal landing zone for the deployment of the SAPIEN valve report favorable outcomes [3, 4, 11]. However, from a technical standpoint, the implantation of self‐expandable valves is inherently more complex due to the extended length of these devices, which must adapt to the intricate and heterogeneous anatomies of outflow tracts. Furthermore, self‐expanding systems may predispose patients to ventricular arrhythmias due to their large “footprint,” which frequently extends into the RVOT [12].

**Figure 6:**
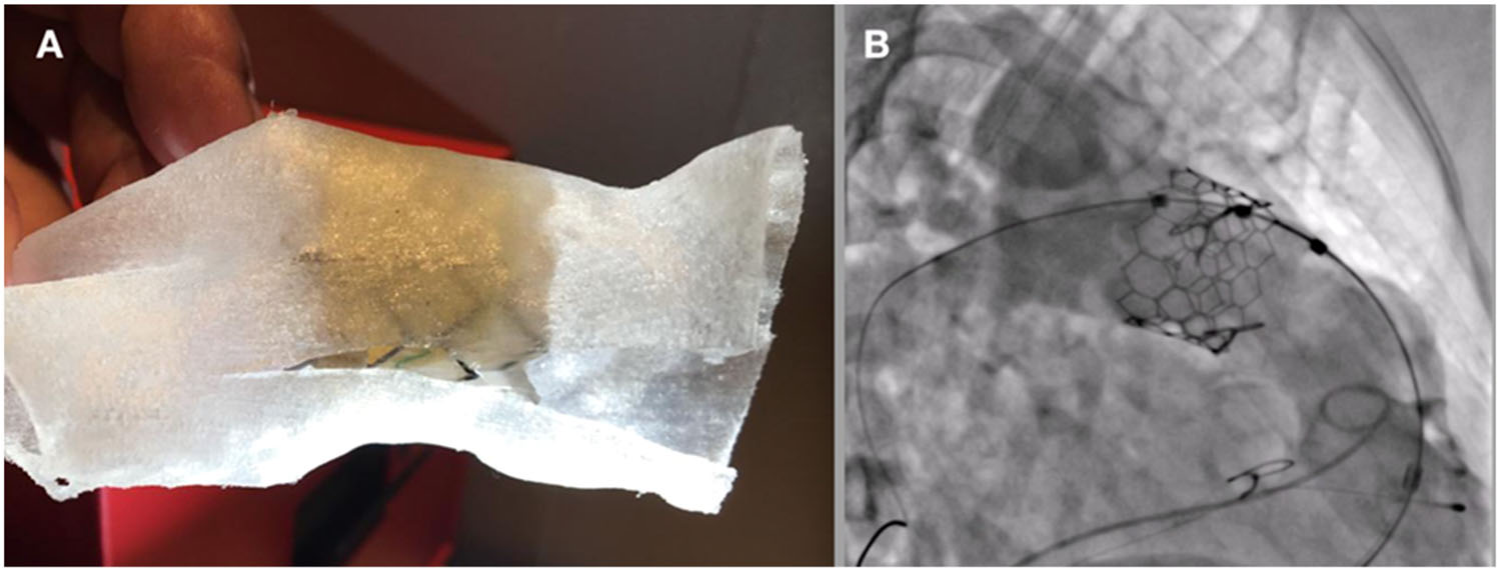
Pre‐implantation computed tomography with three‐dimensional reconstruction and bench test with “in vitro” implantation of a 32‐mm‐diameter valve at the supra‐annular position (A) and post‐implant supra‐annular position with final adequate result (B).

Balloon‐expandable valves > 30 mm have changed the management of patients with complex anatomies [13]. Although technically less demanding for experienced interventional cardiologists due to the design, balloon‐expandable valves carry the inherent risk of embolization, especially in native outflow tracts with varying degrees of distensibility. Pre‐stenting, initially used to prevent Melody™ valve fractures in patients with calcified conduits, is now less common in balloon‐expandable valve implantation, though some operators still use them to reduce embolization risk during surgery [14, 15, 16, 17]. This study emphasizes thorough pre‐ and intra‐procedural evaluations, in some cases using 3D printing of outflow tract models with “in vitro” valve implantation. This approach ensures a safe balloon‐expandable valve implantation landing zone without pre‐stenting (Figure 6).

This report is the largest published experience of balloon‐expandable valve implantation in large RVOTs exceeding 30 mm without pre‐stenting to date. Enlarged native outflow tracts present challenges, especially with balloon‐expandable valves. Furthermore, the presence of various tissue types with different elasticities, including patches, valve rings, native arterial tissue, aneurysmal tissue, infundibular muscle, calcifications, sutures, and fibrotic tissues, further complicate the assessment of outflow tract distensibility and the identification of an optimal location for device positioning and release. On the other hand, self‐expandable valves, due to their shape and length, are more challenging to implant and not suitable for certain anatomies.

Previous studies, including Ogando's 2021 report [18], highlighted the importance of pre‐stent implantation for procedural success with MyVal™ [16, 17]. A recent multicenter experience also recommended pre‐stenting for balloon‐expandable valves in large RVOTs in 80% of patients [13].

In the present study, 17 patients with large outflow tracts underwent MyVal™ implantation. It is important to emphasize the heterogeneity in distensibility observed across outflow tracts in this population, even among those who underwent identical surgical procedures. In light of these findings, we opted to consistently employ noncompliant 30 mm balloons, the largest diameter available in the Brazilian market, inflated to nominal or burst pressure, which we think is crucial in determining the appropriate valve size and ensuring the stability of the implant different from what is recommended when assessing the possibility for self‐expandable valve implantation with compliant balloons [19].

In our initial cases, we considered patients eligible for 32 mm MyVal™ valve implantation when the RVOT did not exceed 31 mm during balloon insufflation. However, as our experience grew, we extended the indication to implant valves even in the absence of a discernible indentation on the balloon and with the final diameter surpassing 31 mm following balloon interrogation. In such cases, a comprehensive coronary compression assessment is important because the valve will be implanted within a larger diameter than that achieved during balloon inflation. In one case, we demonstrated angiographic signs of coronary compression after valve delivery at a diameter approximately 3 mm larger than that demonstrated by the balloon test. This patient has not developed signs of myocardial ischemia, but it corroborates a comprehensive evaluation of the risk of coronary compression in these patients whom severe oversized is performed from balloon interrogation.

Theoretically, pre‐stent deployment reduces the risk of device embolization by establishing a fixed‐diameter landing zone. Nevertheless, the combined use of inflated balloons at nominal or burst pressure, in conjunction with simultaneous ventriculography, ensures achieving the expected final diameter in distensible outflow tracts known to pose challenges in the context of balloon‐expandable valve implantation. This approach avoids the need for stent implantation, thereby reducing associated risks, costs, and potentially the number of procedures a patient must undergo.

## 5 Conclusion

The study demonstrates the feasibility and safety of 30.5 and 32 mm MyVal™ valve implantation in large RVOTs, even in the absence of prior stent placement. All patients had satisfactory valve function immediately after valve implantation. Further studies with larger cohorts and longer follow‐up periods are needed to assess long‐term outcomes, durability, and bacterial endocarditis risk. Its availability in larger diameters expands the treatment options for patients with complex anatomical conditions, providing a new avenue for managing pulmonary insufficiency in this growing population.

## Author Contributions

**João Luiz Langer Manica:** validation, conceptualization, investigation, writing – original draft, writing – review and editing, supervisio, formal analysis, methodology. **Raul Ivo Rossi Filho:** conceptualization, methodology, validation, writing – original draft, investigation. **Carlos Augusto Cardoso Pedra:** conceptualization, writing – original draft, formal analysis, methodology, validation. **Fabio Vieira Caovilla:** methodology, validation, conceptualization, writing – original draft, writing – review and editing, formal analysis. **Francisco Chamié:** conceptualization, investigation, writing – original draft, methodology, validation, formal analysis, writing – review and editing. **Carlo Benatti Pilla:** writing – original draft, writing – review and editing, conceptualization, investigation, validation, methodology, formal analysis. **Pablo Thomé:** conceptualization, investigation, writing – original draft, formal analysis, validation. **Vinicius Fraga:** conceptualization, investigation, validation, writing – review and editing, writing – original draft, formal analysis. **Marcelo Ribeiro:** conceptualization, writing – original draft, validation, formal analysis, methodology. **Germana Cerqueira Coimbra:** conceptualization, writing – original draft, writing – review and editing, validation, formal analysis. **João Henrique Aramayo Rossi:** methodology, validation, investigation. **Ênio Guerios:** conceptualization, investigation, writing – original draft, writing – review and editing, methodology, formal analysis. **João Vitor Slaviero:** conceptualization, investigation, methodology, validation, formal analysis. **Santiago Raul Arrieta:** conceptualization, investigation, writing – original draft, validation, writing – review and editing, formal analysis.

## Conflicts of Interest

The authors declare no conflicts of interest.

## Transparency Statement

The lead author João Luiz Langer Manica affirms that this manuscript is an honest, accurate, and transparent account of the study being reported; that no important aspects of the study have been omitted; and that any discrepancies from the study as planned (and, if relevant, registered) have been explained.

## Data Availability

The data that support the findings of this study are available from the corresponding author upon reasonable request.
